# Core oxidative stress response in *Aspergillus nidulans*

**DOI:** 10.1186/s12864-015-1705-z

**Published:** 2015-06-27

**Authors:** Tamás Emri, Vera Szarvas, Erzsébet Orosz, Károly Antal, HeeSoo Park, Kap-Hoon Han, Jae-Hyuk Yu, István Pócsi

**Affiliations:** Department of Biotechnology and Microbiology, Faculty of Science and Technology, University of Debrecen, P.O. Box 63, H-4032 Debrecen, Hungary; Department of Zoology, Faculty of Sciences, Eszterházy Károly College, Eszterházy út 1, H-3300 Eger, Hungary; Department of Bacteriology, University of Wisconsin, 1550 Linden Dr, Madison, WI 53706 USA; Department of Pharmaceutical Engineering, Woosuk University, 565-701 Wanju, Republic of Korea

**Keywords:** *Aspergillus nidulans*, Oxidative stress, General/environmental stress response, bZIP-type transcription factors, AtfA, Secondary metabolism

## Abstract

**Background:**

The b-Zip transcription factor AtfA plays a key role in regulating stress responses in the filamentous fungus *Aspergillus nidulans*. To identify the core regulons of AtfA, we examined genome-wide expression changes caused by various stresses in the presence/absence of AtfA using *A. nidulans* microarrays. We also intended to address the intriguing question regarding the existence of core environmental stress response in this important model eukaryote.

**Results:**

Examination of the genome wide expression changes caused by five different oxidative stress conditions in wild type and the *atfA* null mutant has identified a significant number of stereotypically regulated genes (Core Oxidative Stress Response genes). The deletion of *atfA* increased the oxidative stress sensitivity of *A. nidulans* and affected mRNA accumulation of several genes under both unstressed and stressed conditions. The numbers of genes under the AtfA control appear to be specific to a stress-type. We also found that both oxidative and salt stresses induced expression of some secondary metabolite gene clusters and the deletion of *atfA* enhanced the stress responsiveness of additional clusters. Moreover, certain clusters were down-regulated by the stresses tested.

**Conclusion:**

Our data suggest that the observed co-regulations were most likely consequences of the overlapping physiological effects of the stressors and not of the existence of a general environmental stress response. The function of AtfA in governing various stress responses is much smaller than anticipated and/or other regulators may play a redundant or overlapping role with AtfA. Both stress inducible and stress repressive regulations of secondary metabolism seem to be frequent features in *A. nidulans*.

**Electronic supplementary material:**

The online version of this article (doi:10.1186/s12864-015-1705-z) contains supplementary material, which is available to authorized users.

## Background

Stress is defined as a change in the environment that results in an internal response in living organisms. All life forms respond to stress, which involves adaptive changes throughout an organism to restore the internal cellular balance in physiological systems. Many aspects of the cellular stress response are not specific to a given stress, as cells monitor stress based on macromolecular damage not the type of stress that causes such damage [1].

The kingdom Fungi have evolved with powerful tools for environmental stress sensing, signaling and adaptation, hence, they can occupy versatile ecological niches [2, 3]. A wealth of information is available on the oxidative stress response systems of the budding yeast *Saccharomyces cerevisiae* [4–7] and the fission yeast *Schizosaccharomyces pombe* [8], which makes the use of yeast-based models popular when the stress response systems of other fungi are studied irrespective of interspecial evolutionary distances [9].

Cross-stress protection phenomena, which prepare fungi to combat more severe forthcoming environmental stress of either the same type or a completely different type, is also wide-spread among fungi [4, 10–14] and obviously contributes to the ecological success of today’s fungi [2, 3].

The molecular mechanism of such cross-protection phenomena may be based on a group of genes co-regulated under various types of environmental stress which are called “Environmental Stress Response” or ESR genes [2, 15]. ESR genes have been identified in *S. cerevisiae* [15], *S. pombe* [16], *Candida albicans* [17] and *Candida glabrata* [18] although their number and regulation varies within an unexpectedly wide spectrum. For example, Msn2/4 C2H2 zinc finger type transcription factors are the master regulators of ESR in both *S. cerevisiae* (size of ESR is ~ 900 genes [15]) and *C. glabrata* (782 genes [18]) meanwhile the bZIP-type (basic leucine zipper DNA interactive domain containing) transcription factor Atf1 [19, 20] is the general ‘all purpose’ regulator of ESR in fission yeast (~140 ESR genes [16]). In *C. albicans*, the existence of ESR was debated [21] but a relatively small group of ESR genes (62 genes) was identified at last under the control of Hog1 mitogen activated protein kinase (MAPK) [17, 22]. Importantly, the way of counting ESR genes has not been standardized yet and seems to be dependent on the dose of stress [17, 21, 22] and the rate of growth [23, 24]. Furthermore, global transcriptional responses to oxidative stress and their regulations were dependent on the type and dose of stress in *S. pombe* [25], and also on the presence or absence of another bZIP-type transcription factor Pcr1, which form heterodimers with Atf1 [26–28].

More recent data published by Berry and Gasch [12] assigned a basically preparative role to ESR against impending stress in budding yeast. Moreover the activation of several different sets of stress response genes by various mild stress conditions may provide budding yeast with satisfactory cross-protection against a given type of severe environmental stress [13]. This observation may help us to explain the general stress tolerance of oxidative stress tolerant strains of *C. albicans* [29] although the size of ESR is small in this opportunistic human pathogen [17].

Interestingly, although it is almost impossible to overestimate the biomedical, economical and ecological significance of the filamentous fungus genus *Aspergillus* [30–35] the information available on the oxidative stress defense systems of these organisms is still limited [9, 36–40], and no data on the existence (or the absence) of *Aspergillus* ESR has been published to the best of our knowledge. A deeper understanding of how the stress response systems of the Aspergilli work may help us, *e.g.* in the identification of novel antifungal drug targets against opportunistic human pathogens like *Aspergillus fumigatus* [40, 41] or in the biological control of mycotoxin productions by toxigenic *Aspergillus* species [42–44].

For our current study, we selected the mycotoxin (*e.g.* sterigmatocystin) producer model organism *Aspergillus nidulans*, which has a complex and robust stress response system incorporating both budding yeast and fission yeast homolog elements, but the overall view is more fission yeast-like [9]. Considering previous literature data, the SakA/HogA MAPK activated AtfA transcription factor, a true functional ortholog of fission yeast’s Atf1, may play a pivotal role in the orchestration of the stress responses of this filamentous fungus [36, 45–49]. Nevertheless, the deletion of *atfA* did not result in any osmotic stress sensitive phenotype in *A. nidulans* [46, 48] but decreased the oxidative stress tolerance of the fungus [48–50]. In spite of the lack of the relevant phenotypes in the gene deletion mutants, AtfA plays an important role in the regulation of global translational changes under osmotic stress, and situates down-stream of SakA/HogA MAPK [47, 49]. In another study, Balázs et al. [48] challenged the hypothesis that a fission yeast-like stress response exists in *A. nidulans* because certain important elements of the oxidative and osmotic stress defense systems were controlled by AtfA but in a stress-specific manner. *E.g. catB* coding for catalase B and *gfdB* encoding glycerol-3-phosphate dehydrogenase B, both under AtfA control, were up-regulated by oxidative (H_2_O_2_ and *t*-butylhydroperoxide; *t*BOOH) and osmotic (NaCl) stress, respectively, but not *vice versa*. It is worth noting that AtfA also possesses important functions in the development of conidiophores [49] and in the stabilization of asexual spores against oxidative and heat stress [46]. Importantly, other Atf1 (and AtfA) homologs with significant physiological functions have been characterized more recently in other Aspergilli including *Aspergillus oryzae* (AtfA and AtfB [51, 52]) and *Aspergillus fumigatus* (AtfA [53, 54]) and in other ascomycetes, for example *Claviceps purpurea* (CPTF1 [55], *Neuropsora crassa* (ATF-1 [56]), *Magnaporthe oryzae* (Moatf1 [57]), *Fusarium graminearum* (Fgatf1 [58]), *Fusarium oxysporum* f. sp. *cubense* (Foatf1 [59]) and *Penicillium marneffei* (AtfA [60]), which gives further actuality to this study.

Considering previous literature data on *A. nidulans* AtfA, we aimed at (i) recording and comparing global transcriptional changes in *A. nidulans ΔatfA* gene deletion mutant and control cultures exposed to various types and doses of oxidative stress and also to osmotic stress using a whole-genome-based 60-mere DNA microarray [61], (ii) comparing these transcriptome data with the results of previous EST-based DNA microarray [36] and proteome [62] studies, (iii) defining a group of genes co-regulated under oxidative stress (Core Oxidative Stress Response or COSR genes) or under environmental stress (ESR genes), (iv) estimating the importance of AtfA transcription factor under unstressed conditions and in the regulation of COSR and ESR and (v) analyzing the impact of various stress conditions and *atfA* deletion on the regulation of various secondary metabolite gene clusters.

## Results

**Fig. 1:**
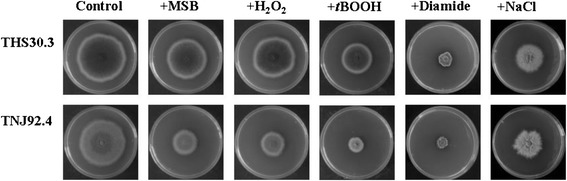
Comparison of the stress sensitivities of control and *ΔatfA Aspergillus nidulans* strains. Plates were point-inoculated with freshly grown conidia (10^5^ conidia in 5 μl aliquots of 0.9 % NaCl, 0.01 % Tween 80) and were incubated at 37 °C for 5 d (Yin et al., 2013 [63]). All assays were carried out in triplicates, and representative photos are presented here. The stress sensitivites of the *ΔatfA* strain were always higher than those of the control at any concentrations of the oyidative stress generating agents tested. In contrast, there was no difference between the relative growths of the mutant and control strains when exposed to NaCl. The employed stressor concentrations were: *t*BOOH: 0.8 mM, H_2_O_2_: 6.0 mM, MSB: 0.12 mM, diamide: 2.0 mM and NaCl: 0.6 M. Representative photos are presented

An *A. nidulans ΔatfA* gene deletion strain and the appropriate control strain were generated. The *ΔatfA* mutant was more sensitive to menadione sodium bisulfite (MSB), H_2_O_2_, *t*-butylhydroperoxide (*t*BOOH), diamide (but not to NaCl) than the control strain (Fig. 1, Table 1) at all stressor concentrations tested, which was in agreement with previous observations by Balázs et al. [48].

**Table 1:** Stress sensitivity of the control and the ΔatfA strains in surface cultures

Growth^a^	Stress exposures				
	MSB (0.12 mM)	H_2_O_2_ (6 mM)	*t*BOOH (0.8 mM)	Diamide (2 mM)	NaCl (0.6 M)
Colony diameter (%) (control strain)	82 ± 3	84 ± 6	70 ± 8	34 ± 3	56 ± 4
Colony diameter (%) (*ΔatfA* strain)	52 ± 3*	54 ± 6*	41 ± 6*	26 ± 4*	57 ± 4

*Significant difference between the two strains was demonstrated using the Student’s t-test (p < 0.05)

^a^Growth was characterized with the diameter of the colonies recorded at 5 d. Colony diameters are given in the percentage of the colony diameter measured in untreated cultures. Mean ± S.D. values, calculated from 4 independent experiments are presented

Genome-wide expression changes caused by different stress conditions were studied in *ΔatfA* and control strains. The applied stressor concentrations (0.12 mM MSB, 5 and 75 mM H_2_O_2_, 0.8 mM tBOOH, 1.8 mM diamide and 0.6 M NaCl) were comparable to those used previously by Hagiwara et al. [46], Lara-Rojas et al. [49] and Yin et al. [63]. The strains continued their growths in the presence of the stress generating agents as presented in Table 2. Interestingly, a full recovery in growth was reached in 10 h after H_2_O_2_ exposures (both 5 and 75 mM) in the control strain meanwhile a lower biomass production was recorded in cultures containing MSB, diamide or NaCl (appr. 22.5-35.5 % less biomass in comparison to untreated control). A more significant growth reduction was only recorded in cultures exposed to *t*BOOH (appr. 61.3 % less biomass). Importantly, per cent growth reductions found in *ΔatfA* cultures (full recovery for l-H_2_O_2_, appr. 24.1-34.5 % growth reductions for MSB, diamide and NaCl, and 72.4 % less biomass for *t*BOOH treated cultures) were quite similar to those found in control cultures with the exception of 75 mM H_2_O_2_, where a 34.5 % growth reduction indicated a somewhat slower recovery than that observed in h-H_2_O_2_-exposed control cultures (Table 2). Taking into consideration these growth reductions, *t*BOOH. MSB, diamide and NaCl treatments were regarded “severe” stress conditions meanwhile H_2_O_2_-initiated stress was considered “mild” (at least for the control strain).

**Table 2:** Increases in the dry cell mass (DCM) values determined under various stress conditions in cultures of the control and the ΔatfA strains

Biomass gains^a^	Stress exposures^b^						
	No stress	MSB	l-H_2_O_2_	h-H_2_O_2_	*t*BOOH	Diamide	NaCl
ΔDCM (g l^−1^)^2^ (control strain)	3.1 ± 0.4	2.1 ± 0.3*	3.1 ± 0.4	3.0 ± 0.5	1.2 ± 0.2*	2.4 ± 0.3*	2.0 ± 0.3*
ΔDCM (g l^−1^)^2^ (*ΔatfA*)	2.9 ± 0.4	2.2 ± 0.3*	2.8 ± 0.3	1.9 ± 0.3*	0.8 ± 0.1*	2.2 ± 0.3*	1.9 ± 0.2*

*Significant difference between untreated and treated cultures was demonstrated using the Student’s t-test (p < 0.05)

^a^Changes in dry cell mass (DCM) values were recorded 10 h after stress treatments. Mean ± S.D. values, calculated from 4 independent experiments are presented

^b^The following stressor concentrations were employed in these experiments: MSB: 0.12 mM, low concentration (l)-H_2_O_2_: 5 mM, high concentration (h)-H_2_O_2_: 75 mM, *t*BOOH: 0.8 mM, diamide: 1.8 mM and NaCl: 0.6 M

Importantly, good correlations with correlation coefficients between 0.71-0.88 were found between DNA chip-based gene expression data and the qRT-PCR-based gene expression validation data gained with 67 gene specific primer pairs (Additional file 1: Table S1). On the other hand, we found only weak correlations with correlation coefficients of pairwise comparisons less than 0.2 (data not shown) between current gene expression data sets and the data coming from our earlier study, which was based on DNA microarrays containing 3533 unique PCR-amplified probes [36]. However, some co-regulated genes were identified, which are summarized in Additional file 2: Table S2 and which include AN2846 (*gpxA*) putative glutathione peroxidase gene (up-regulated after 0.5 h MSB and diamide treatments) as well as the AN1182 (*benA*) ß-tubulin, AN6632 putative 40S ribosomal protein and AN6181 putative 60S ribosomal protein genes, all of which were down-regulated after 0.5 h MSB treatments in both studies.

We also compared the new whole-genome-based DNA chip data with our previous proteome data set [62], and it was found that out of the 153 stress-responsive proteins identified in 0.8 mM MSB treated cultures at 6 h exposure time 59 (39 %) behaved in line with the gene expression changes recorded in this study (with at least four-times changes in their transcriptions) meanwhile in the case of 35 (23 %) proteins the proteome and transcriptome level changes were significant but just in the opposite sense (data not shown). Some notable co-regulated genes are summarized in Additional file 3: Table S3 and include AN2846 (*gpxA*), encoding a putative glutathione peroxidase, AN3581 (*trxR*), coding for a putative thioredoxin reductase, which stress response elements were up-regulated at the levels of both transcription and translation after MSB exposures as well as AN10223, a putative peroxiredoxin gene and AN7388 (*catD*), a putative catalase-peroxidase gene, which genes and also their protein products were equally down-regulated under MSB treatments.

### COSR and cross-stress adaptations

“Severe” oxidative stress conditions generated by MSB, *t*BOOH, diamide (but not by H_2_O_2_) exposures caused up-regulation or down-regulation of more than 1000 genes each in both the control and the *ΔatfA* gene deletion strains (Table 3). In contrast, “mild” stress initiated by H_2_O_2_ (5 and 75 mM) as well as by NaCl (high-osmolarity stress) had typically less effect on the transcriptome with less than or around 1000 genes up-regulated or down-regulated (Tables 3 and 4). The numbers of co-regulated genes in different stress-exposed control and *ΔatfA* cultures indicate clearly that the more stressors were included in the comparison of the global transcriptional changes the less co-regulated genes were found (Tables 3 and 4).

**Table 3:** Number of co-regulated genes in stress-exposed control and ΔatfA cultures

Transcriptional changes^a^	Stress exposures^b^						
	MSB	MSB *t*BOOH	MSB *t*BOOH diamide	MSB *t*BOOH diamide + h-H_2_O_2_	MSB *t*BOOH diamide + NaCl	MSB *t*BOOH diamide + h-H_2_O_2_ and NaCl	All^c^
THS30.3 control strain							
*Up-regulated genes*							
>2x increases in 6 stresses							7
>2x increases in 5 stresses						51	88
>2x increases in 4 stresses				161	84	226	221
>2x increases in 3 stresses			393	414	503	535	523
>2x increases in 2 stresses		699	793	856	1031	1012	1004
>2x increase in 1 stress	1574	1542	2052	2018	2007	1925	1923
Sum:	1574	2241	3238	3449	3625	3749	3766
	MSB only	*t*BOOH only	Diamide only	h-H_2_O_2_ only	NaCl only		l-H_2_O_2_ only
>2x increases	1574	1366	1877	799	1097		153
*Down-regulated genes*							
>2x decreases in 6 stresses							6
>2x decreases in 5 stresses						65	84
>2x decreases in 4 stresses				103	194	278	300
>2x decreases in 3 stresses			480	530	544	543	580
>2x decreases in 2 stresses		786	960	1021	985	1031	1069
>2x decrease in 1 stress	1696	1563	1799	1904	1813	1852	1763
Sum:	1696	2349	3239	3558	3536	3769	3802
	MSB only	*t*BOOH only	Diamide only	h-H_2_O_2_ only	NaCl only		l-H_2_O_2_ only
>2x decreases	1696	1441	2022	789	1032		317
TNJ 92.4 *ΔatfA* strain							
*Up-regulated genes*							
>2x increases in 6 stresses							8
>2x increases in 5 stresses						39	63
>2x increases in 4 stresses				192	69	233	235
>2x increases in 3 stresses			289	331	379	387	399
>2x increases in 2 stresses		561	850	834	951	939	1006
>2x increase in 1 stress	1001	1781	2103	2104	2138	2141	2149
Sum:	1001	2342	3242	3461	3534	3739	3860
	MSB only	*t*BOOH only	Diamide only	h-H_2_O_2_ only	NaCl only		l-H_2_O_2_ only
>2x increases	1001	1902	1767	863	774		354
*Down-regulated genes*							
>2x decreases in 6 stresses							10
>2x decreases in 5 stresses						111	123
>2x decreases in 4 stresses				290	163	289	310
>2x decreases in 3 stresses			440	360	552	531	546
>2x decreases in 2 stresses		740	918	882	850	779	823
>2x decrease in 1 stress	1050	1354	1523	1510	1516	1497	1489
Sum:	1050	2094	2881	3042	3081	3207	3301
	MSB only	*t*BOOH only	Diamide only	h-H_2_O_2_ only	NaCl only		l-H_2_O_2_ only
>2x decreases	1050	1784	1845	835	845		329

^a^Numbers of genes with at least two-fold increase or decrease in their transcriptions under the specified stress conditions are presented for both the control and the *ΔatfA* strains

^b^Stressor concentrations are presented in footnotes to Table 2

^c^MSB, *t*BOOH, diamide, l-H_2_O_2_, h-H_2_O_2_ and NaCl stress treatments

**Table 4:** Number of co-regulated genes in H_2_O_2_-exposed control and ΔatfA cultures

Transcriptional changes^a^	Strain					
	THS30.3 control strain			TNJ 92.4 *ΔatfA* strain		
	Up-regulated genes	Down-regulated genes	Up- or down-regulated genes	Up-regulated genes	Down-regulated genes	Up- or down-regulated genes
l-H_2_O_2_	153	317	470	354	329	683
h-H_2_O_2_	799	789	1588	863	835	1698
at least in one of them	833	835	1668	1050	999	2049
in both of them^b^	119 (0.78)	271 (0.85)	390 (0.83)	167* (0.47)	165* (0.50)	332* (0.49)

*The number of genes regulated in both H_2_O_2_ experiments in respect to the number of genes regulated by l-H_2_O_2_ treatment is significantly smaller in the *ΔatfA* mutant than in the control strain according to the Fisher’s exact test (p < 0.05)

^a^Numbers of genes with at least two-fold increase or decrease in their transcriptions under the specified stress conditions are presented for both the control and the *ΔatfA* strains. Stressor concentrations are presented in footnotes to Table 2

^b^Numbers of genes regulated in both experiments are given. The number of genes regulated in both experiment/number of genes regulated in the presence of l-H_2_O_2_ ratios are also given in parenthesis

**Fig. 2:**
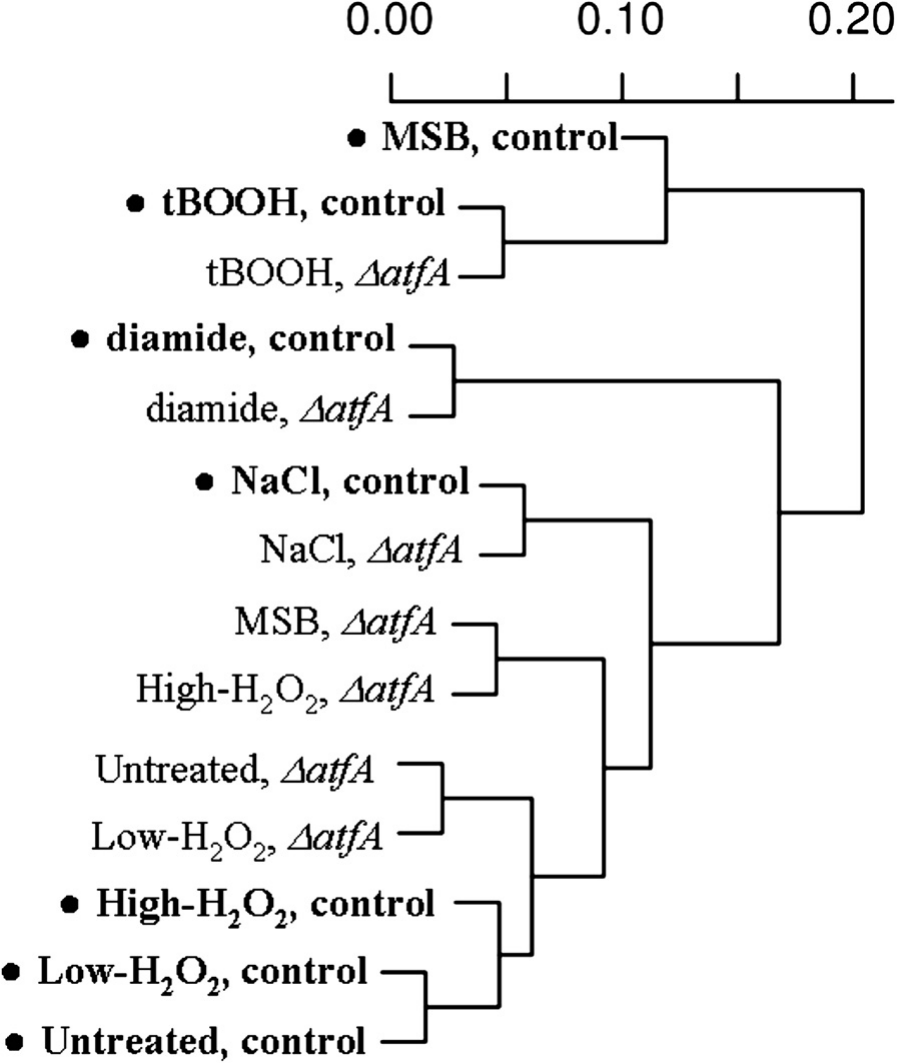
Comparison of transcriptome data sets of stress-exposed control and *ΔatfA* strains. Pairwise similarities between transcriptome profiles were characterized by absolute correlations of normalized microarray data presented in Additional file 4: Table S4, and are summarized using agglomerative hierarchical cluster analysis with complete linkage

COSR genes were defined as genes showing unidirectional transcriptional changes under the three “severe” oxidative stress conditions tested (stress conditions generated by MSB, *t*BOOH and diamide). Relying on these premises, the numbers of COSR genes were 873 and 729 in the control and *ΔatfA* strains, respectively (Table 3). The subsequent inclusion of either h-H_2_O_2_ or NaCl or even concomitantly both stress treatment data sets into the comparative analysis of the transcriptional changes reduced markedly the number of core stress response genes (Table 3). The inclusion of all stress treatments (l-H_2_O_2_, h-H_2_O_2_ and also NaCl) almost completely emptied the group of core stress response genes; only 13 and 18 genes (Table 3) showed unidirectional transcriptional changes in all stress exposure experiments in the control strain and the *ΔatfA* strain, respectively. The pairwise similarities between transcriptome profiles (Fig. 2, Additional file 4: Table S4) as well as the great number of genes showing transcriptional changes only in one stress treatment (Table 3) also suggested that the different stressors had characteristic stress-specific effects on the transcriptome.

Regarding the two H_2_O_2_ stress treatments, the majority (78–85 %) of genes regulated by l-H_2_O_2_ exposure were also regulated by h-H_2_O_2_ exposure in the control strain (Table 4). Interestingly, the overlap between the effects of the two treatments was significantly smaller (47–50 %) in case of the *ΔatfA* strain (Table 4).

A significant portion of COSR genes was uncharacterized and more than 50 % of the COSR genes in the control strain did not appear in the group of COSR genes defined in the *ΔatfA* mutant (Additional file 5: Table S5). For example, *napA* (AN7513) coding for a bZIP-type oxidative stress response regulatory transcription factor [50] was only part of the COSR genes in the THS30.3 control strain meanwhile *rsmA* (AN4562), encoding another bZIP-type transcription factor involved in the regulation of secondary metabolism [63], was a COSR gene in both the control and the *ΔatfA* mutant strains concomitantly (Additional file 5: Table S5). Induction of *rsmA* in both the control and the mutant strains as well as a significantly higher induction of *napA* in the control than in the mutant strain under MSB, tBOOH and diamide stress treatments was also verified by qRT-PCR experiments (Additional file 1: Table S1).

In order to find significant shared GO terms among those systematically used to describe COSR genes, the GO Term Finder of Aspergillus Genome Database (http://www.aspergillusgenome.org) was used. Importantly, no significant shared GO term was found in the group of genes incorporating up-regulated COSR genes in the control strain. On the other hand, several GO terms related to cell cycle (*e.g.* replication, cytoskeleton functions, nuclear and cell division) as well as the GO terms “ribosome biogenesis” and “sterol metabolic process” were significantly shared in the group of down-regulated COSR genes (Additional file 6: Table S6). In contrast, the GO term “cell redox homeostasis” and several other GO terms related to ribosome biogenesis were significantly shared in the sets of up-regulated and down-regulated genes, respectively, in the *ΔatfA* gene deletion strain (Additional file 6: Table S6).

**Fig. 3:**
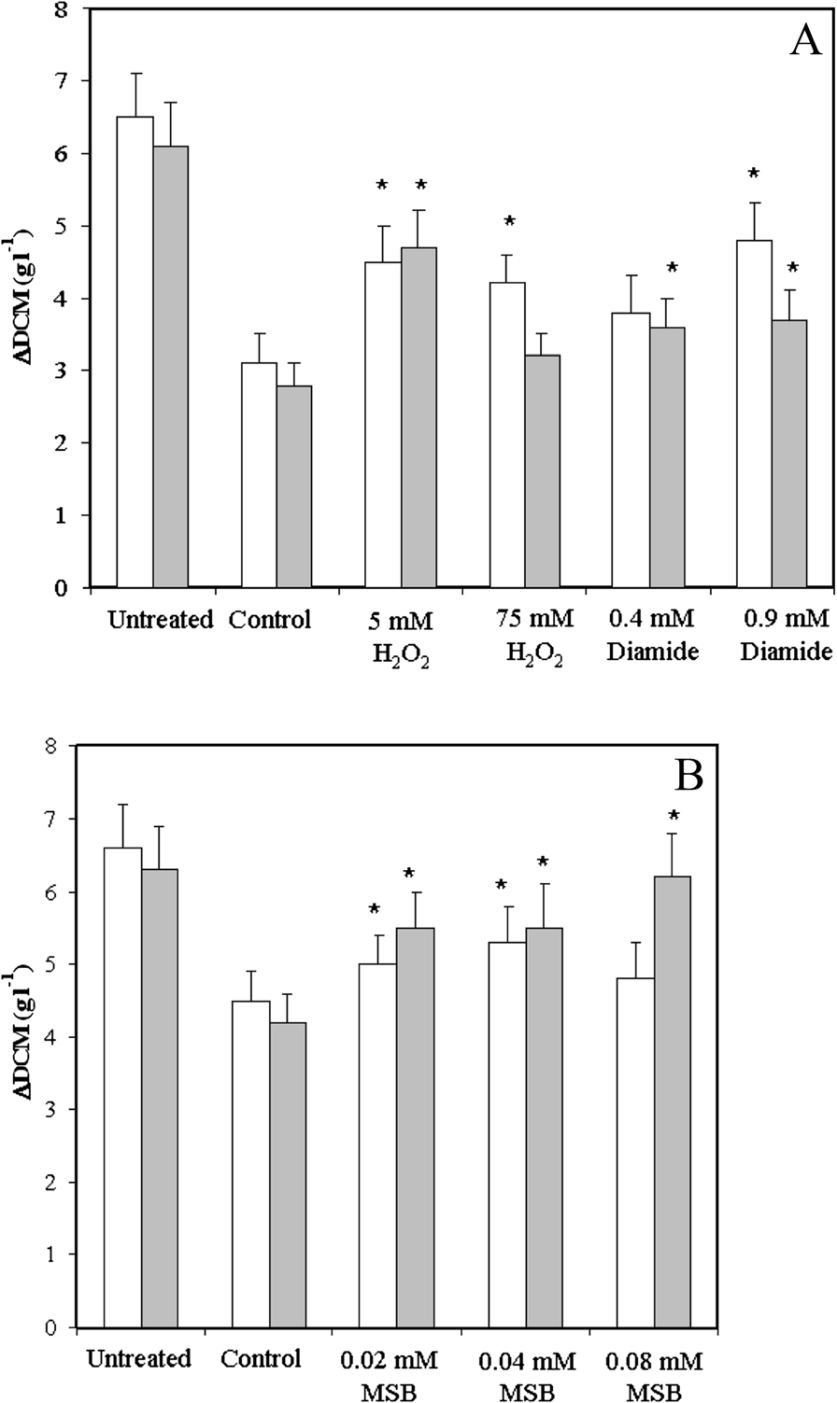
Cross adaptations observed in the control and *ΔatfA* strains. The control (white columns) and *ΔatfA* (grey columns) strains were pre-cultured for 0.5 h in the presence of various stress initiating agents as indicated. Following pre-treatments, fungi were exposed to 0.18 mM MSB (Part **a**) or 1.0 M NaCl (Part **b**) in fresh culture media. All cultures were incubated for 18 h, and increases in the dry cell mass (DCM) values were determined, which are presented here as means ± S.D. values (n = 3). Untreated cultures were not subjected to stress treatments at all meanwhile control cultures were exposed to MSB and NaCl without stress pre-treatments. *Significant differences in comparison to control cultures calculated by Student’s t-test (p < 0.05, n = 3)

Although only few core stress response genes (Table 3) were found in both the control and the *ΔatfA* strains the stress responses still shared some common motives when the physiological functions of the up-regulated and down-regulated genes were compared. Among the significant shared GO terms “sterol metabolic process” (MSB, l-H_2_O_2_, h- H_2_O_2_, tBOOH and diamide) and “ribosome biogenesis” (MSB, h- H_2_O_2_, tBOOH, diamide and NaCl) were characteristic for down regulated gene, meanwhile “branched-chain amino acid biosynthetic process” (MSB, h- H_2_O_2_, tBOOH) and “fatty acid catabolic process” (tBOOH, diamide and NaCl) for up-regulated genes under stress treatments indicated in parentheses (data not shown). Cross-stress adaptation experiments demonstrated that pre-treatments of the cultures with low concentrations of H_2_O_2_ or diamide decreased the growth inhibitory effects of MSB and pre-treatments with a low concentration of MSB decreased the growth inhibitory effect of NaCl in both the control and the *ΔatfA* strains (Fig. 3).

### Consequences of atfA gene deletion

Deletion of *atfA* altered the transcriptome profile of *A. nidulans* under unstressed conditions (Fig. 2, Additional file 4: Table S4). In the untreated cultures, 657 genes showed up-regulation while 542 were down-regulated in comparison to the control strain (Table 5).

**Table 5:** Effect of atfA deletion on the transcriptome under different stress conditions

	Stress exposures^a^						
	None	MSB	l-H_2_O_2_	h-H_2_O_2_	tBOOH	Diamide	NaCl
	Number of genes influenced by stress exposure and/or *atfA* deletion						
Increased transcription							
Only in control^b^	-	1053	117	444	391	497	709
Only in *ΔatfA* ^c^	-	480	318	508	927	387	386
In both strains^d^	-	521	36	355	975	1380	388
Control vs. *ΔatfA* ^e^	657	1646	649	585	709	526	725
AtfA-dependent^f^	657	721	64	130	148	60	313
Ratio of AtfA-dependent genes^g^		0.46	0.42	0.16	0.11	0.03	0.29
Decreased transcription							
Only in control^h^	-	1228	189	467	395	696	653
Only in *ΔatfA* ^i^	-	582	201	513	738	519	466
In both strains^j^	-	468	128	322	1046	1326	379
Control vs. *ΔatfA* ^k^	542	1815	648	661	749	310	431
AtfA-dependent^l^	542	857	34	74	91	64	119
Ratio of AtfA-dependent genes		0.51	0.11	0.09	0.06	0.03	0.12

^a^Stressor concentrations are presented in footnotes to Table 2

^b^Up-regulation was defined by the threshold of log_2_ (I_treated, control_/I_untreated, control_) > 1 and log_2_ (I_treated, *ΔatfA*_/I_untreated, *ΔatfA*_) ≤ 1

^c^Up-regulation was defined by the threshold of log_2_ (I_treated, *ΔatfA*_/I_untreated, *ΔatfA*_) > 1 and log_2_ (I_treated, control_/I_untreated, control_) ≤ 1

^d^Up-regulation was defined by the threshold of log_2_ (I_treated, control_/I_untreated, control_) > 1 and log_2_ (I_treated, *ΔatfA*_/I_untreated, *ΔatfA*_) > 1

^e^Up-regulation was defined by the threshold of log_2_ (I_treated, control_ /I_treated, Δ*atfA*_) > 1

^f^Up-regulation was defined by the threshold of log_2_ (I_treated, control_ /I_treated, Δ*atfA*_) > 1, log_2_ (I_treated, control_/I_untreated, control_) > 1 and log_2_ (I_treated, *ΔatfA*_/I_untreated, *ΔatfA*_) ≤ 1

^g^Number of AtfA dependent genes/number of genes regulated in the control strain

^h^Down-regulation was defined by the threshold of log_2_ (I_treated, control_/I_untreated, control_) < −1 and log_2_ (I_treated, *ΔatfA*_/I_untreated, *ΔatfA*_) ≥ −1

^i^Down-regulation was defined by the threshold of log_2_ (I_treated, *ΔatfA*_/I_untreated, *ΔatfA*_) < −1 and log_2_ (I_treated, control_/I_untreated, control_) ≥ −1

^j^Down-regulation was defined by the threshold of log_2_ (I_treated, control_/I_untreated, control_) < −1 and log_2_ (I_treated, *ΔatfA*_/I_untreated, *ΔatfA*_) < −1

^k^Down-regulation was defined by the threshold of log_2_ (I_treated, control_ /I_treated, Δ*atfA*_) < −1

^l^Down-regulation was defined by the threshold of log_2_ (I_treated, control_ /I_treated, Δ*atfA*_) < −1, log_2_ (I_treated, control_/I_untreated, control_) < −1 and log_2_ (I_treated, *ΔatfA*_/I_untreated, *ΔatfA*_) ≥ −1

In the gene deletion mutant, GO terms related to secondary metabolism enriched significantly for up-regulated genes (Additional file 7: Table S7). The significant shared GO terms for down-regulated genes included the followings: „cell wall organization”, “conidiophore development”, “phosphorelay signal transduction system”, “regulation of protein phosphorylation”, “calcium ion transmembrane transport” as well as “response to stimulus”. The latter GO term is used for the description of a group of genes coding for important elements of the stress response system including *catB*, *trxR*, AN1131, encoding a putative cytosolic Cu/Zn superoxide dismutase and *msnA*, encoding a transcription factor involved in the regulation of oxidative and salt stress responses [64–66] (Additional file 7: Table S7).

Deletion of *atfA* also altered the transcription profile of a large group of genes under various stress conditions (Table 5). The lack of the AtfA transcription factor prevented or reduced markedly the induction or repression of many genes which were stress-responsive in the control strain (Table 5; see “only in control” genes). In this group of genes, only those were regarded as AtfA-dependent where these transcriptional effects attributed to *atfA* deletion resulted in at least 2-fold differences in the gene expression levels recorded in the control and gene deletion strains under stress treatments (Table 5; see the legend of “AtfA-dependent” genes either) The *atfA* gene deletion also resulted in up-regulation or down-regulation of certain genes which were not stress-responsive in the control strain (Table 5; see “only in *ΔatfA*” genes).

Deletion of *atfA* caused the largest size transcriptional changes in the MSB-treated cultures (Table 5, Fig. 2 and Additional file 4: Table S4). The ratio of AtfA-dependent, up-regulated genes and stress-responsive, up-regulated genes was also high in the case of l-H_2_O_2_ and NaCl treatments (Table 5). In contrast, *atfA* deletion affected only slightly the genome-wide transcriptional changes during diamide and tBOOH treatments (Table 5; Fig. 2 and Additional file 4: Table S4). In fact, the highest correlation between the transcriptome profiles of the *ΔatfA* and control strains was found between the diamide treated cultures (Fig. 2, Additional file 4: Table S4).

The overlap between the groups of the AtfA-dependent genes found under various stress treatments was negligible. Importantly, among the COSR genes (Additional file 5: Table S5) only two were AtfA-dependent: AN7823, a gene in the sterigmatocystin gene cluster and encoding a putative peroxidase and AN9451, which is a function-unknown gene. Furthermore, there was no overlap between the significant shared GO terms recoded in the groups of AtfA-dependent genes under various stress conditions (data not shown).

### Secondary metabolism and stress

Various stress conditions also influenced significantly the secondary metabolism of *A. nidulans* (Table 6, Additional files 8 and 9: Tables S8, S9). Among the 155 secondary metabolite biosynthesis genes up-regulated in the control strain 29 genes encoded transcription factors, non-ribosomal peptide synthases, polyketide synthases or prenyltransferases, which were designated as “key genes” in further analyses. In many cases, only one or two genes showed significant transcriptional changes within one gene cluster. Therefore, only a cluster where more than half of its genes including at least one of its “key genes” were up-regulated was regarded as an up-regulated cluster. Altogether 5 clusters (clusters AN7884, AN6236, AN1680, AN10486 as well as the derivative of the benzaldehyde and F9775 hybrid cluster 2) showed up-regulation in the control strain at least in one stress treatment (Additional files 8 and 9: Tables S8 and S9). It is notable, that the numbers of down-regulated and up-regulated secondary metabolite biosynthetic genes, secondary metabolism key genes and secondary metabolite gene clusters were comparable (Table 6). The clusters with stress-dependent down-regulation included the emericellamide cluster, clusters AN2924, AN8209 and AN7838 (AN12331), the microperfuranone cluster, the AN6236 cluster as well as the “no PKS/NRPS backbone 4” cluster (Additional files 8 and 9: Tables S8 and S9).

**Table 6:** Stress dependent regulation of secondary metabolite genes and clusters

	Stress exposures^a^							
	None	MSB	L-H_2_O_2_	H-H_2_O_2_	tBOOH	Diamide	NaCl	All
THS30.3 control strain								
*Up-regulation*								
Key genes^b^	-	11	2**	5	16	16	4	29
All genes^c^	-	65	17**	25**	65	71	34**	155
Clusters^d^	-	2	0	0	3	1	0	5
*Down-regulation*								
Key genes	-	11	3	8	8	8	9	19
All genes	-	46	22	37	38^***^	52	39	112
Clusters	-	5	1	0	1	3	2	7
TNJ 92.4 *ΔatfA* strain								
*Up-regulation*								
Key genes	-	16	15*	16*	11	11	1	42
All genes	-	53	48*	68*	60	54	29	179
Clusters	-	2	3	2	2	0	0	7
*Down-regulation*								
Key genes	-	5***	3***	5***	19*	20*	17***	31
All genes	-	16*^,^***	22***	26***	71*	82*	70*^,^***	139
Clusters	-	0	1	2	5	7***	5	9
TNJ 92.4 (*ΔatfA*) vs. THS30.3 (control)								
*Up-regulation*								
Key genes	11							
All genes	43							
Clusters	4							
*Down-regulation*								
Key genes	5							
All genes	22***							
Clusters	0							

*Significant difference between the THS30.3 control and TNJ 92.4 (*ΔatfA*) strain according to the Fisher’s exact test (p < 0.05; n_key genes_ = 94, n_all genes_ = 467, n_clusters_ = 66)

**Significant difference in comparison to MSB, tBOOH and diamide treatment as well (in the same row) according to the Fisher’s exact test (p < 0.05; n_key genes_ = 94, n_all genes_ = 467, n_clusters_ = 66)

***Significant difference between the up- and down-regulated genes according to the Fisher’s exact test (p < 0.05; n_key genes_ = 94, n_all genes_ = 467, n_clusters_ = 66)

^a^Stressor concentrations are presented in footnotes to Table 2

^b^Key genes were defined as secondary metabolite cluster genes encoding transcription factors, non-ribosomal peptide synthases, polyketide synthases, terpene synthase or prenyltransferases according to Inglis et al. [114]

^c^Only genes of clusters determined either manually or experimentally were involved in the analysis [114]

^d^Clusters were regarded as up-regulated (or down-regulated) cluster if at least one of its key genes and more than half of its manually or experimentally determined genes were up-regulated (down-regulated)

Deletion of *atfA* increased significantly the number of stress-inducible secondary metabolite biosynthetic genes and “key genes” under l-H_2_O_2_ and h-H_2_O_2_ exposures meanwhile decreased significantly the number of such stress-responsive genes in the case of diamide and *t*BOOH stress (Table 6, Additional files 8 and 9: Tables S8, S9). It is noteworthy that three secondary metabolite gene clusters, namely the monodictyphenone cluster, the derivative of the benzaldehyde and F9775 hybrid cluster 1 and the pkf cluster were up-regulated in the *ΔatfA* mutant in comparison to the control strain even under unstressed conditions and were also up-regulated under l-H_2_O_2_ and/or h-H_2_O_2_ exposures and were down-regulated when the gene deletion strain was exposed to diamide (not in the case of the monodictyphenone cluster), *t*BOOH or NaCl stress (Additional files 8 and 9: Tables S8 and S9). Up-regulation of *pkeA* (encoding the polyketide synthase of the benzaldehyde and F9775 hybrid cluster 1), *pkfA* (encoding the polyketide synthase of pkf cluster) and *mdpG* (encoding the polyketide synthase of the monodictyphenone cluster) in the *ΔatfA* mutant in comparison to the control strain under unstressed conditions and under h-H_2_O_2_ exposures, as well as down-regulation of *pkeA* and *pkfA* n the mutant strain under tBOOH and diamide stress were validated by qRT-PCR experiments (Additional file 1: Table S1).

## Discussion

Oxidative and salt stress induced genome-wide transcriptional changes in *A. nidulans*, which were highly depended on the type and strength of the stress (Tables 3 and 4, Fig. 2). The observed global stress responses were similar to those found by other researchers earlier. For example, cell division-related processes, which are decisively important in the maintenance of vegetative growth and which influence replication, transcription, translation and cytoskeleton functions as well as sterol metabolism were inhibited under severe stress conditions in this study (Additional file 6: Table S6) as well as in other previous studies and in other fungi [7, 8, 36, 67, 68]. Up-regulation of catalase and peroxidase genes, furthermore genes coding for elements of glutathione, thioredoxin and trehalose metabolisms as well as for heat shock proteins and parts of DNA repair (Additional file 1: Table S1) have also been observed by other researchers in various fungal species [7, 8, 36, 69, 70].

On the other hand, only weak correlation was found between the current gene expression data sets and the data coming from our earlier transcriptome [36] and proteome [62] studies. Besides of the variations in the culture and stress conditions applied in these works, the weak correlation can be clearly explained by several other important differences between the stress conditions employed. In the recent study, we used a whole-genome-based DNA chip with 60-mer oligonucleotide probes designed to reduce cross-hybridizations between probes. In previous experiments by Pócsi et al. [36], an expressed sequence tag based DNA was applied, and in this case, potential cross-hybridization between paralogue genes can be a serious problem [71]. It is also worth mentioning that the FGSC26 strain used in our previous study, in contrast to strains used recently, harbored *biA1* (biotin auxotrophy) and *veA1* mutations [36]. Recent studies demonstrated that VeA is important in the regulation of oxidative stress response in several fungi including *A. flavus* [72], *Cochliobolus heterostrophus* [73] and *Botrytis cinerea* [74]. Moreover, nutritional supplements, *e.g.* riboflavin, paba, pyridoxine, can also have an impact on the observed stress sensitivity of the strains tested [48]. Although biotin did not affect significantly the growth of *A. nidulans* [48] the presence of this supplement in the medium may have influenced the stress response at the level of transcriptome. Poor correlation is a common problem when proteome and transcriptome data are compared, and the majority of the differences can be explained with divergent post-transcriptional and post-translational regulations [75]. Considering all these variations in the experimental arrangements, it is remarkable that certain genes, notably AN2846 (*gpxA*) encoding a putative glutathione peroxidase and AN3581 (*trxR*) coding for thioredoxin reductase [76], still showed steady up-regulation independently of the applied strains and methods (Additional files 2 and 3: Tables S2 and S3). Up-regulation of these genes seems to be crucial during MSB induced stress, which demonstrates the paramount importance of both the glutathione-based and the thioredoxin-based elements of the oxidative stress defense system in *A. nidulans* [36, 76, 77].

### Environmental Stress Response (ESR) and COSR

ESR was first defined in the budding yeast *Saccharomyces cerevisiae* as a sum of stereotypical changes observable in the transcription of more than 900 genes in response to very different types of stress [15, 78]. In their study, Gasch et al. [15] found approximately 300 genes up-regulated and 600 genes down-regulated in more than 20 different stresses in baker’s yeast. Later, Chen et al. [16] studied global gene expression changes under five stress conditions (heat, H_2_O_2_, Cd^2+^, sorbitol and methylmethane sulfonate stress) in the fission yeast *Schizosaccharomyces pombe*, and demonstrated that approximately 140 genes showed more than two-fold increases in their transcription in at least four stresses and approximately 100 genes showed more than two-fold decreases in their expression in at least three stresses [16]. Importantly, the number of stress specific genes induced by only one stressor was less than 100 in each case [16]. In the present study, the number of co-regulated genes (merely 7 genes co-induced and 6 genes co-repressed under all the five oxidative stress conditions as well as under NaCl exposures, and when l-H_2_O_2_ treatments were omitted from analyses 51 + 65 genes showed co-regulation (Table 3). Meanwhile the number of co-regulated genes was small the number of genes regulated exclusively by one certain type of stress was well above 1000 (Table 3). These observations together with the sharply decreasing number of co-regulated genes as a function of the number of stress initiating agents studied (Table 3) does not support the existence of a *S. cerevisiae*-type ESR in *A. nidulans*.

We assume that the observed co-regulations were most likely consequences of the overlapping physiological effects of the stressors especially in the case of severe stress treatments and not of the existence of a general ESR. Severe stress causes aspecific damages in versatile biomolecules like proteins, nucleic acids and lipids, decreases the ATP/AMP ratio or influences the redox balance and ion homeostasis independently of the way of the initiation of stress. Such non-specific physiological changes may be reflected, at least to some extent, in the stress-initiated alterations in the transcriptome profiles.

On the other hand, comparing stress treatments similar in type and strength can be a useful and beneficial strategy to identify a group of genes co-regulated by the same stress sensing and signaling pathways. COSR gene groups were constructed by identifying and collecting co-regulated genes through mapping the global transcriptional changes recorded under three “severe” oxidative stress conditions elicited by MSB, *t*BOOH and diamide treatments (Additional file 5: Table S5). Based on this experimental arrangement, COSR genes were found in great number (873 genes) and, similarly to the ESR genes in *S. cerevisiae* [15, 78], the function of the up-regulated genes was very diverse with no significant shared GO term identified meanwhile the majority of the down-regulated genes was related to the maintenance of vegetative growth, *e.g.* replication, cytoskeleton functions as well as nuclear and cell divisions. Further studies are needed to identify the stress signaling and regulatory pathways governing the expression of the COSR genes. It is noteworthy that two bZIP-type transcription factors, NapA and RsmA, are transcriptionally regulated within the frame of COSR in *A. nidulans* (Additional files 1 and 5: Tables S1 and S5), which suggests the importance of both the maintenance of the redox homeostasis of the cells and the production of secondary metabolites [50, 63] as an inseparable part of the oxidative stress defense.

The characteristics of ESRs observed in various fungi are summarized in Table 7. Unfortunately, the experimental design (*e.g.* the type, the strength and the number of the tested stresses as well as the criteria used to define stereotypical changes) was different in these experiments, which is a limitation when we compare the data. However, Table 7 suggests that ESR may be limited only to the budding yeast *S. cerevisiae* and to its close relatives like *C. glabrata*, where Msn2/4 transcription factors evolved to regulate stress responses under a wide spectrum of environmental stress. It is noteworthy that Msn2/4 regulate numerous, but not all, genes up-regulated in ESR, and these transcription factors are probably not involved in down-regulations [15]. Roetzer et al. [18] found only limited overlap (268 genes) between the ESRs of *S. cerevisiae* and *C. glabrata*, and this overlap is even smaller when other species are considered [16, 17]. These data question the existence of a universal stress-response set of genes in fungi, the induction of which were equally beneficial in all fungal species and in all ecological niches they occupy. Fungi seem to choose one of two options, evolving a set of ‘unique’ stress responses or, instead, a ‘general’ stress response. A set of stress-specific “unique” stress responses can provide the fungus with an appropriate adaptation to a wide array of stress but need numerous genes and a complex and robust signaling network to regulate them like that described in the a Aspergilli [9, 48, 79, 80]. On the other hand, a general stress response can be operated well even with less genes and with a less complex signaling network and can provide the fungi like saccharomycetous yeasts with a perhaps less sophisticated but instantaneous stress response even to cope with impending stress [12, 13]. Importantly, the number of *S. cerevisiae* genes is approximately half of that of the Aspergilli, which indicates that the type of stress response (“unique” *vs.* “general”) is likely also dependent on the size of the fungal genome (Table 7).

**Table 7:** Properties of ESR in different fungi

Species	Number of genes^a^	Number of genes showing stereotypical behavior in different stresses	Regulator of ESR	Reference
*Saccharomyces cerevisiae*	5907 (WGD^b^)	868	Msn2/4	[15]
*Candida glabrata*	5214 (WGD)	752	Msn2/4	[18]
*Schizosaccharomyces pombe*	5123	140	Sty1	[16]
*Candida albicans*	6219	61	Hog1	[17]
*Aspergillus nidulans*	10678	116	?	This study

^a^The numbers of protein encoding genes were originated from the following web pages: http://www.ncbi.nlm.nih.gov, http://www.pombase.org, http://www.candidagenome.org, http://www.aspgd.org

^b^WGD: whole genome duplication

A wide spectrum of genetic evidence demonstrates that overexpression of even a single gene can increase the stress tolerance [81, 82] and, therefore, if this gene is part of ESR its up-regulation by one stress can cause adaptation to another stress [16, 78]. This explanation is commonly used to explain cross-stress adaptation phenomena and the physiological significance of ESR. Cross-stress adaptation was also observed in both the control and the *ΔatfA* strains in our experiments (Fig. 3). The most interesting cross-stress adaptation was developed with H_2_O_2_ when employed at 5 mM concentration, which alone caused only small transcriptional changes in *A. nidulans* and these alterations in gene expressions were quite different from those caused by MSB (Fig. 2; only 81 up-regulated and 24 down-regulated genes overlapped). Importantly, l-H_2_O_2_ exposures did not elicit even two-fold increases in the transcriptions of genes encoding basically important elements of MSB stress response, including FeS cluster assembly and DNA repair proteins, trehalose, glutathione or thioredoxin metabolic and antioxidant enzymes as well as heat shock proteins and metallo-chaperones (data not shown). However, using 5 mM H_2_O_2_ in stress pre-treatments resulted in clear-cut adaptation to severe MSB stress (Fig. 3). In accordance with these observations, Berry et al. [13] were able to induce H_2_O_2_ tolerance in *S. cerevisiae* by pre-exposing baker’s yeast cultures to mild NaCl, dithiotreitol or heat stress although there were only little overlaps in the lists of genes induced by different pre-treatments. In another study, Guan et al. [83] found that Ctt1 catalase produced under NaCl pre-treatment was distributed to daughter cells during subsequent divisions and was responsible for the elevated H_2_O_2_ tolerance of *S. cerevisiae* cells. They also demonstrated that stress pre-treatments caused a faster response in gene expression during subsequent high-dose stress treatments, which required the nuclear pore component protein Nup42 [83]. Furthermore, several studies have demonstrated the overlapping nature of stress signaling pathways with numerous interplays, co-operations and even cross-talks between them [8, 79, 80]. The regulation of this complex and robust network is based on protein-protein interactions and/or modifications rather than on transcriptional changes alone. A possible explanation for cross-stress adaptation therefore is that various pre-treatments can activate the signaling network, which increases subsequently the efficiency of sensing of and/or responding to versatile types of environmental stress. It is possible that changes in the expressions of stress response genes during pre-treatments contribute to the adaptation to impending, more severe environmental stress. However, we suggest that transcriptional up-regulations of stress response genes under pre-treatments are not essential to reach cross-stress adaptations in *A. nidulans*.

### Involvement of AtfA in the regulation of stress response in *A. nidulans*

Atf1 is a bZIP-type transcription factor regulated by the Sty1 MAPK pathway in *S. pombe* and is responsible for regulation of genes involved in various stress responses including heat, oxidative, reductive, osmotic and starvation stress [84]. Atf1 can form heterodimer with another bZIP-type transcription factor, Pcr1 and some of the target genes are regulated by this hetrodimer [85]. In *A. nidulans*, the Atf1 orthologue AtfA is regulated by the HogA/SakA MAPK pathway [49]. The phenotypes of the *ΔatfA* gene deletion strains demonstrate that AtfA is necessary for normal vegetative growth and sporulation as well as for oxidative and heat stress tolerance in *A. nidulans* [46–49] (Fig. 1, Table 1). In the present study, the deletion of *atfA* affected the transcription of an unexpectedly high number of genes under MSB stress (Fig. 2, Table 5). In contrast, the transcriptome profiles of the *ΔatfA* mutant and the control strains were more similar during H_2_O_2_, *t*BOOH, NaCl and especially under diamide treatments (Fig. 2, Table 5). Moreover, the lack of *atfA* also affected the transcription of several genes under unstressed conditions. Deletion of *atf1* in *S. pombe* also caused inductions and repressions of several genes even in unstressed cultures and also prevented the induction of numerous genes under stress treatments [16].

The stress-dose dependent activation of Atf1 is also well described in *S. pombe.* Atf1 regulates the oxidative stress response in high dose H_2_O_2_ treatments while its importance is less significant when fission yeast was exposed to low H_2_O_2_ concentrations when the Pap1 transcription factor played a key role [86]. In *A. nidulans*, the ratio of AtfA-dependent genes was much higher in l-H_2_O_2_ (5 mM) elicited stress than in h-H_2_O_2_ (75 mM) triggered stress (Table 5). However the overlap between the two stress responses was significantly less in the *ΔatfA* mutant than in the control strain (Table 4). These observations suggest that the majority of AtfA-dependent genes in l-H_2_O_2_ stress were also part of the h-H_2_O_2_ stress response. In fact, 77 out of the 98 AtfA-dependent genes recorded in l-H_2_O_2_ stress also showed stress responsive regulation under h-H_2_O_2_ exposures. Therefore, the low ratio of the AtfA-dependent genes during h-H_2_O_2_ elicited stress was not the consequence of the decreased number of AtfA-dependent genes but could be attributed to the increased number of AtfA-independent genes instead.

One of the main differences between the regulations of the oxidative stress responses in *A. nidulans* and *S. pombe* is that the number of genes likely under AtfA control was more stress-type-dependent in *A. nidulans* than in *S. pombe* (Table 5). Several genes showing AtfA-dependent regulation in one stress treatment did not show any AtfA-dependency under another stress condition in our experiments. It is remarkable that even in the group of the COSR genes merely two showed an AtfA-dependent regulatory pattern (Additional file 5: Table S5).

Several GO terms related to stress signaling and regulation (“phosphorelay signal transduction system”, “regulation of protein phosphorylation”, “calcium ion transmembrane transport” as well as “response to stimulus”) were typical of the group of down-regulated genes in the *ΔatfA* mutant under unstressed conditions (Additional file 7: Table S7). According to this, we hypothesize that AtfA coordinates the up-regulation of certain regulatory genes (e.g. members of the phosphorelay signal transduction system; Additional file 7: Table S7) under environmental stress and also determined their basal transcription levels under unstressed conditions. Decreases in the expressions of these genes in the gene deletion mutant grown in unstressed cultures disturbed the homeostasis of the strain, resulted in alterations in the transcription patterns of a large number of other genes (Fig. 2, Table 5, Additional file 7: Table S7).

Due to the networking nature of signaling pathway, the missing AtfA was compensated by other regulatory proteins under H_2_O_2_, tBOOH, diamide, NaCl, which resulted in global transcriptional profiles very similar to those recorded for the control strain (Fig. 2, Table 5). Considering a most recently published study of Bok et al. [50] the transcription factor NapA (orthologue of *S. pombe* Pap1 [86]), another bZIP-type oxidative stress response regulator, can be a candidate which may take over AtfA functions under hydrogen peroxide induced oxidative stress. NapA, which is under RsrA control, seems to be the master regulator of the specific response to peroxide stress [50].

When cells were exposed to MSB the signaling network was unable to substitute AtfA satisfactorily, which resulted in serious disturbances in the cell homeostasis and concurrently altered the transcription levels of a large group of genes, which were therefore described as potential AtfA targets (Fig. 2, Table 5). Further research is needed to identify which genes among the AtfA target genes responsible for the efficient stress response under MSB stress treatment.

### Secondary metabolism and stress response

Emerging data demonstrate that there are interplays between the regulations of oxidative stress response and secondary metabolism in fungi. Induction of secondary metabolite production by oxidative stress and its inhibition by antioxidants have been observed in several species, and even transcription factors affecting both secondary metabolism and oxidative stress response have been identified [42–44, 63, 66, 87–90]. Our transcriptome data also support the importance of stress in the regulation of secondary metabolism because all stressors including NaCl affected significantly the transcription of secondary metabolite biosynthesis genes (Table 6, Additional file 8: Table S8) under experimental conditions like culturing at 37 °C in glucose containing minimal medium, which are generally not beneficial for secondary metabolite production in this species. Moreover, the *ΔatfA* strain, in addition to being more sensitive to oxidative stress (Fig. 1), also had a more altered expression pattern of secondary metabolism genes under various stress treatments when compared to the control strain (Table 6, Additional files 8 and 9: Tables S8 and S9).

Both *rsmA* (AN4562) and *napA* (AN7513) was part of the COSR in the control strain (Addtional file 5: Table S5) which is in good accordance with increased number of up-regulated secondary metabolit genes a secondary metabolit key genes under MSB, *t*BOOH and diamide stress in comparison to the other stress applied (Table 6). It supports the view that RsmA and NapA can be a link between the regulation of stress response and secondary metabolite production in *A. nidulans* [43, 63, 89].

Cryptic secondary metabolite gene clusters are in the center of industrial investigations since they may be exploitable in the production of novel secondary metabolites. In addition to the overexpression of the complete gene cluster in a suitable organism [91], the overexpression of a cluster-specific transcription factor or, alternatively, a global regulator of secondary metabolism, *e.g.* LaeA, in the host organism [92, 93] are frequently used techniques to identify the products of cryptic gene clusters. Our study demonstrate that deletion of an oxidative stress response regulator gene in combination with mild oxidative stress can also be applicable to overproduce the products of certain gene clusters, which may lead to identification of new secondary metabolites (Additional file 9: Table S9).

It is worth noting that many known secondary metabolite gene clusters of *A. nidulans* were not stress responsive in our experiments (Additional files 9 and 10: Tables S9 and S10). Since we studied only the early global transcriptional changes further studies will address the question if the regulation of these clusters and genes are independent of environmental stress or the progression of the transcriptional changes will need more time. More importantly, environmental stress cannot only induce some secondary metabolite gene clusters but can also repress others (Table 6, Additional file 9: Table S9). Strategies based on the application of certain antioxidants to prevent the accumulation of ROS and, consequently, the formation of mycotoxins may efficiently inhibit the production of certain mycotoxins but, concomitantly, may also induce the formation of other unwanted secondary metabolites.

The ecological and/or physiological value of the redox regulation of secondary metabolite production is unclear. Reverberi et al. [42] suggested that production at least some the secondary metabolites (*e.g.* aflatoxins) contained several oxidative steps therefore their biosynthesis helped maintaining the redox status of the cells under oxidative stress. On the other hand, beside well-known and well-characterized ROS productions observable during pathogen – host interactions, competing micro-organisms can also elicit oxidative stress either through the extracellular formation of H_2_O_2_ [94, 95] or *via do novo* synthesis of secondary metabolites, which generate oxidative stress in sensitive organisms [96–98]. Such ecological role can be attributed for example to aflatoxin B1 produced by certain aspergilli [99]. Similarly, hyperosmotic stress is also frequently induced *e.g.* by ethanol producing microbes [100]. The “artificial” stressors used in research laboratories may imitate the attack of a competitive species or a host organism, which may explain the stress dependent regulation of secondary metabolite clusters. Other explanations, like the use of secondary metabolite spectra to inform other cells of the same species about the physiological status of a given cell cannot be ruled out either. Connections between secondary metabolite production and development as well as the importance of secondary metabolites or secondary metabolite-like compounds in the regulation and coordination of sporulation, germination or sexual development of a colony have been demonstrated by several researchers [101–103].

## Conclusions

*A. nidulans* showed very stress-specific (in case of MSB stress highly AtfA-dependent) global transcriptional stress responses under the stress conditions tested in this study. The remarkable flexibility of the stress response system operating in *A. nidulans*, and probably in other aspergilli as well, explains the evolutionary success of this genus. This flexibility can be important for human pathogenic species, *e.g.* for *A. fumigatus*, to cope with the harsh and stressful environmental conditions present in the human body and, meanwhile, it can also set up challenges when we aim at the improvement of industrial strains. The stress-dependent regulation of secondary metabolite gene clusters is of paramount importance when cryptic secondary metabolites are identified and also when novel strategies to control the production of secondary metabolites including mycotoxins are considered and evaluated.

## Methods

### Strains, culture conditions, stress sensitivity tests and cross-stress adaptation experiments

In this study, *A. nidulans* TNJ 92.4 (*pyrG89*, *AfupyrG*^+^; *pyroA4*; *ΔatfA::pyroA*; *veA*^+^) as a *ΔatfA* gene deletion strain and THS30.3 (*pyrG89*, *AfupyrG*^+^; *pyroA*^+^; *veA*^+^) as the appropriate control strain were used. The *atfA* deletion (∆*atfA*) mutants were generated by double-joint PCR (DJ-PCR) as described [104]. The flanking regions of each *atfA* gene were amplified by PCR with primer pair, oNK-968 (5’-AGTTGCGTCATCACGTTATTGGTG-3’), 974(5’-ACTTCTGCAGTCGGAATTGGCCTGATGGGTGGCACACAACCATAGATC-3’) (*atfA*5’ with *pyroA* tail) and oNK-971 (5’-ACTACCTTTGATGAGTGCTGACTAG-3’), 975 (5’-TGGTGAGAACACATGCACAACTTGATCACTTGACTAACTGGCGCAACG-3’) (*atfA*3’ with *pyroA* tail) from the genomic DNA. The *pyroA* marker was amplified with the primer pair oNK-395 (5’-ATCTCATGGGTGCTGTGCGAAAGG-3’):oNK-396 (5’-TTGCATCGCATAGCATTGCATTGC-3’). The final deletion construct was amplified with the nested primer set oNK-972 (5’-TGCAGAGACTTCAAGAGTCAAGAG-3’):oNK-973 (5’-TACACATCTGCCATGACATCTCTG-3’) and was introduced into TNJ 36 (*pyrG89*, *AfupyrG+*; *pyroA4*; *veA*^+^) [105] using the Vinoflow FCE lysing enzyme (Novo Nordisk) [106]. For THS30.3, the partial wild type *pyroA*^+^ PCR fragment covering the *pyroA4* mutation was amplified with the primer pair oHS656 (5’-GGACCCGAGAGGCGAGAGCTTA-3’):oHS657 (5’-GACACCATCACAGCCAAGCTGC-3’) from the genomic DNA and was introduced into TNJ 36. The strains were maintained on Barratt’s nitrate minimal medium (NMM medium [107]), and NMM agar plates were incubated at 37 °C for 6 d [63]. Conidia harvested from these 6-day-old plates were used in all further experiments.

The stress sensitivities of the strains were tested on NMM agar plates containing 0.03-0.24 mM MSB, 2–12 mM H_2_O_2_, 0.2-1.2 mM *t*BOOH, 0.5-4 mM diamide or 0.5-2 M NaCl. Plates were spot-inoculated with 5 μl freshly made conidia suspension (10^5^ conidia ml^−1^) and were incubated at 37 °C for 5 d. Diameters of the colonies were measured and used for the characterization of the stress sensitivities of the strains [63].

To study genome-wide transcriptional changes, fungi were grown in shake flasks (500 ml) containing 100 ml Barratt’s NMM broth. All submerged cultivations were carried out at 37 °C and at 3.7 Hz shaking frequency. Cultures were inoculated with 1x10^8^ conidia and were incubated for 16 h. Three parallel cultures of the control strain were mixed and than were divided into three equal, 100–100 ml, parts. In the case of the *ΔatfA* strain, nine 16 h cultures were mixed, the mycelial pellets were let sink down and appr. 600 ml supernatant was removed. The remaining 300 ml cell suspension containing now the vegetative fungal tissue from nine cultures was divided into three equal, 100–100 ml, parts. As a result, the starting dry cell masses (DCMs) were always between 4–5 g l^−1^ for both the mutant and the control strains. In stress exposure experiments, the cultures were treated with 0.12 mM MSB, 5 and 75 mM H_2_O_2_, 0.8 mM *t*BOOH, 1.8 mM diamide, 0.6 M NaCl or were kept untreated (control), and were further incubated for 0.5 h. The growth inhibitory effects of the different stressors were estimated by measuring the reduction in the increase of DCM at 10 h after treatment according to Pusztahelyi et al. [108].

In cross-stress adaptation experiments, 16 h cultures were pre-treated with 0.02, 0.04 and 0.08 mM MSB, 5 and 75 mM H_2_O_2_, 0.4 and 0.9 mM diamide or were kept untreated (control), and were further incubated for 0.5 h as described above. After stress pre-treatments, mycelia were filtered out on sintered glass, were washed with freshly prepared NMM medium and were transferred immediately into fresh NMM medium also supplemented with 0.18 mM MSB or 1 M NaCl. Increases in the DCMs were detected after cultivation for 18 h.

### Quantitative real-time reverse-transcription polymerase chain reaction (qRT-PCR) assays

Samples were always taken at 30 min after stress treatment. Total RNA was isolated from lyophilized mycelia coming from 4 parallel experiments following the instructions of Chomczynski [109]. qRT-PCR experiments were carried out as described earlier [110] with the primers and annealing temperatures presented in Additional file 10: Table S10. Primers were designed based on the locus sequences of *A. nidulans* FGSC A4 obtained from The Broad Institute’s homepage (www.broadinstitute.org). Relative transcription levels were quantified with ΔΔCP = ΔCP_treated_ – ΔCP_control_, where ΔCP_treated_ = CP_reference gene_ − CP_tested gene_ measured from treated cultures or the cultures of the mutant strain, ΔCP_control_ = CP_reference gene_ - CP_tested gene_ measured from untreated cultures or the wild type strain. CP values stand for the qRT-PCR cycle numbers of crossing points. The *actA* gene (AN6542 [79]) was used as reference gene.

### Microarray analysis

For DNS chip studies, Agilent 60-mer oligonucleotide high density arrays 4 × 44 K (Kromat Ltd., Budapest, Hungary) were constructed. Oligos were designed with the eArray software of Agilent (design number 031140). Total RNA was isolated from lyophilized mycelia after 30 min after stress treatments as described above in qRT-PCR assays. Importantly, total RNA pools used in microarray and qRT-PCR analyses were isolated in independent experiments. For DNA chip studies, RNA samples gained from three parallel experiments were pooled in 1:1:1 ratios.

Cyanine-3 (Cy3) labeled cRNA was prepared according to Agilent’s One-Color Microarray-Based Gene Expression Analysis Low Input Quick Amp Labeling protocol, followed by RNeasy Mini spin column purification (QIAGEN). The quality of labeled cRNA was evaluated on the Agilent Bioanalyser 2100 and quantified using an ND-1000 NanoDrop spectrophotometer. Fragmented cRNA samples (1650 ng; specific activity >20.0 pmol Cy3/μg cRNA) were applied to the individual arrays. Slides were placed into a rotating Agilent hybridization oven and were hybridized at 65 °C and 10 rpm for 17 h. After hybridization, microarrays were washed at room temperature with GE Wash Buffer 1 (Agilent) and at 37 °C GE Wash buffer 2 (Agilent), then dried by brief centrifugation. Slides were scanned immediately after washing on the Agilent DNA Microarray Scanner (FE SW 11.1) using one color scan setting for 1x44k array slides (Extended Dynamic Range, Scan Area 61×21.6 mm, Scan resolution 5 μm, Dye channel is set to Green and Green PMT is set to XDR Hi 100 % and XDR Lo 10 %). Agilent’s Feature Extraction software (version 11.1) was used to obtain prenormalised data. Prenormalised microarray data (median foreground, median background) was background corrected using the normexp + offset method suggested by Ritchie et al. [111] followed by quantile normalization between arrays [112] as in Smyth [113]. The full data set was deposited in the Gene Expression Omnibus (GEO; http://www.ncbi.nlm.nih.gov/geo/) with the following accession number: GSE63019.

Genes showing at least two-time-increase or decrease in their relative transcription levels were regarded as up-regulated and down-regulated genes, respectively. Physiological function categories were created according to the GO annotation available at AspGD (http://www.aspergillusgenome.org) and at Broad Institute (http://www.broadinstitute.org). In the case of the secondary metabolite genes, cluster borders determined either manually or experimentally were used [114]. The GO Term Finder of Aspergillus Genome Database (http://www.aspergillusgenome.org) was used to find significant shared GO terms of those used to describe the genes in a selected list (p < 0.1).

### Calculating similarities between transcriptome data sets

Pairwise similarities between global transcription profiles were measured by absolute correlations of normalized microarray data and summarized using agglomerative hierarchical cluster analysis with complete linkage using the R 2.15.2 software [115].

## Additional files

Additional file 1: Table S1. Transcriptional changes of selected genes measured with qRT-PCR. Additional file 2: Table S2. Comparison of old and new transcriptome data sets. Additional file 3: Table S3. Comparison of proteome and new transcriptome data sets. Additional file 4: Table S4. Pairwise correlation coefficients of transcriptome data sets. Additional file 5: Table S5. List of COSR genes. Additional file 6: Table S6. List of significant shared GO terms for COSR genes. Additional file 7: Table S7. List of AtfA dependent genes in untreated cultures as well as list of significant shared GO terms for them. Additional file 8: Table S8. Up- and down-regulated secondary metabolite genes. Additional file 9: Table S9. Stress dependent behavior of secondary metabolism gene clusters in *A. nidulans*. Additional file 10: Table S10. Primer pairs used in this study.

## Abbreviations

COSR: Core Oxidative Stress Response
DCM: Dry cell mass
ESR: Environmental stress response
MSB: Menadione sodium bisulfite
NMM medium: Barratt’s nitrate minimal medium
*t*BOOH: *t*-butylhydroperoxide
