# Supplementary material for: Core oxidative stress response in Aspergillus nidulans
Source: BMC Genomics. 2015 Jun 27;16(1):478. doi: 10.1186/s12864-015-1705-z (PMC4482186; doi:10.1186/s12864-015-1705-z)
Supplement: Additional file 4: Table S4. — Pairwise correlation coefficients of transcriptome data sets. [file 12864_2015_1705_MOESM4_ESM.doc]

**Supplementary Table 4** Pairwise correlation coefficients of transcriptome data sets

Pairwise similarities between transcription profiles were measured by absolute correlations of normalized microarray data.

|  |  | Control strain | | | | | | | *ΔatfA* strain | | | | | | |
| --- | --- | --- | --- | --- | --- | --- | --- | --- | --- | --- | --- | --- | --- | --- | --- |
|  |  | MSB | tBOOH | Diamide | NaCl | h-H_2_O_2_ | l-H_2_O_2_ | Untreated | MSB | tBOOH | Diamide | NaCl | h-H_2_O_2_ | l-H_2_O_2_ | Untreated |
| Control strain | MSB | **1.000** | 0.897 | 0.849 | 0.846 | 0.860 | 0.854 | 0.867 | 0.877 | 0.881 | 0.822 | 0.809 | 0.848 | 0.796 | 0.821 |
|  | tBOOH | 0.897 | **1.000** | 0.875 | 0.873 | 0.910 | 0.893 | 0.894 | 0.920 | 0.951 | 0.858 | 0.863 | 0.903 | 0.848 | 0.859 |
|  | Diamide | 0.849 | 0.875 | **1.000** | 0.857 | 0.865 | 0.854 | 0.864 | 0.879 | 0.877 | 0.973 | 0.853 | 0.855 | 0.832 | 0.856 |
|  | NaCl | 0.846 | 0.873 | 0.857 | **1.000** | 0.935 | 0.928 | 0.928 | 0.903 | 0.851 | 0.856 | 0.942 | 0.888 | 0.889 | 0.909 |
|  | h-H_2_O_2_ | 0.860 | 0.910 | 0.865 | 0.935 | **1.000** | 0.977 | 0.953 | 0.932 | 0.861 | 0.865 | 0.927 | 0.949 | 0.939 | 0.943 |
|  | l-H_2_O_2_ | 0.854 | 0.893 | 0.854 | 0.928 | 0.977 | **1.000** | 0.985 | 0.914 | 0.851 | 0.853 | 0.934 | 0.923 | 0.951 | 0.958 |
|  | Untreated | 0.867 | 0.894 | 0.864 | 0.928 | 0.953 | 0.985 | **1.000** | 0.920 | 0.861 | 0.853 | 0.930 | 0.908 | 0.941 | 0.961 |
| *ΔatfA* strain | MSB | 0.877 | 0.920 | 0.879 | 0.903 | 0.932 | 0.914 | 0.920 | **1.000** | 0.902 | 0.882 | 0.912 | 0.954 | 0.923 | 0.937 |
|  | tBOOH | 0.881 | 0.951 | 0.877 | 0.851 | 0.861 | 0.851 | 0.861 | 0.902 | **1.000** | 0.883 | 0.869 | 0.879 | 0.822 | 0.852 |
|  | Diamide | 0.822 | 0.858 | 0.973 | 0.856 | 0.865 | 0.853 | 0.853 | 0.882 | 0.883 | **1.000** | 0.888 | 0.876 | 0.848 | 0.874 |
|  | NaCl | 0.809 | 0.863 | 0.853 | 0.942 | 0.927 | 0.934 | 0.930 | 0.912 | 0.869 | 0.888 | **1.000** | 0.919 | 0.927 | 0.948 |
|  | h-H_2_O_2_ | 0.848 | 0.903 | 0.855 | 0.888 | 0.949 | 0.923 | 0.908 | 0.954 | 0.879 | 0.876 | 0.919 | **1.000** | 0.951 | 0.947 |
|  | l-H_2_O_2_ | 0.796 | 0.848 | 0.832 | 0.889 | 0.939 | 0.951 | 0.941 | 0.923 | 0.822 | 0.848 | 0.927 | 0.951 | **1.000** | 0.978 |
|  | Untreated | 0.821 | 0.859 | 0.856 | 0.909 | 0.943 | 0.958 | 0.961 | 0.937 | 0.852 | 0.874 | 0.948 | 0.947 | 0.978 | **1.000** |
