# Supplementary material for: Core oxidative stress response in Aspergillus nidulans
Source: BMC Genomics. 2015 Jun 27;16(1):478. doi: 10.1186/s12864-015-1705-z (PMC4482186; doi:10.1186/s12864-015-1705-z)
Supplement: Additional file 9: Table S9. — Stress dependent behavior of secondary metabolism gene clusters in A. nidulans. [file 12864_2015_1705_MOESM9_ESM.doc]

**Supplementary Table 9** Stress dependent behavior of secondary metabolism gene clusters in *A. nidulans*

| **Cluster** | **Size^1^** | **Regulated genes** | **Stress dependence^2^** | **Regulated key genes** |
| --- | --- | --- | --- | --- |
| AN7884 cluster | 15  (14 on chip) | 9 | up-regulation in MSB stress (control) | AN7884 (NRPS), AN7872 (TF) |
|  |  | 7 | up-regulation in MSB stress (*ΔatfA*) | AN7872 (TF) |
|  |  |  |  |  |
| Monodictyphenone (mdp) cluster | 12  (11 on chip) | 6 | up-regulation after *atfA* deletion | AN0150 (PKS), AN0148 (TF) |
|  |  | 11 | up-regulation in l-H_2_O_2_ stress (*ΔatfA*) | AN0150 (PKS), AN0148 (TF) |
|  |  | 10 | up-regulation in h-H_2_O_2_ stress (*ΔatfA*) | AN0150 (PKS), AN0148 (TF) |
|  |  | 8 | down-regulated in tBOOH stress (*ΔatfA*) | AN0150 (PKS), AN0148 (TF) |
|  |  | 8 | down-regulated in NaCl stress (*ΔatfA*) | AN0150 (PKS) |
|  |  |  |  |  |
| Derivative of Benzaldehyde1 (dba) and F9775 hybrid cluster 2^3^ | 10 | 6 | up-regulation in tBOOH (control) | AN7909 (PKS) |
|  |  |  |  |  |
| Derivative of Benzaldehyde1 (dba) and F9775 hybrid cluster 1 | 9 | 7 | up-regulation after *atfA* deletion | AN7903 (PKS), AN7896 (TF) |
|  |  | 5 | up-regulation in h-H_2_O_2_ stress (*ΔatfA*) | AN7903 (PKS), AN7896 (TF) |
|  |  | 6 | down-regulated in tBOOH stress (*ΔatfA*) | AN7903 (PKS), AN7896 (TF) |
|  |  | 6 | down-regulated in diamide stress (*ΔatfA*) | AN7903 (PKS), AN7896 (TF) |
|  |  | 6 | down-regulated in NaCl stress (*ΔatfA*) | AN7903 (PKS), AN7896 (TF) |
|  |  |  |  |  |
| pkf cluster^3^ | 6 | 6 | up-regulation after *atfA* deletion | AN3230 (PKS) |
|  |  | 4 | up-regulation in l-H_2_O_2_ stress (*ΔatfA*) | AN3230 (PKS) |
|  |  | 4 | down-regulated in tBOOH stress (*ΔatfA*) | AN3230 (PKS) |
|  |  | 5 | down-regulated in diamide stress (*ΔatfA*) | AN3230 (PKS) |
|  |  | 5 | down-regulated in NaCl stress (*ΔatfA*) | AN3230 (PKS) |
|  |  |  |  |  |
| Emericellamide (eas) cluster^3^ | 5 | 3 | down-regulated in MSB stress (control) | AN2545 (NRPS), AN2547 (PKS) |
|  |  | 3 | down-regulated in tBOOH stress (control) | AN2545 (NRPS), AN2547 (PKS) |
|  |  | 3 | down-regulated in diamide stress (control) | AN2545 (NRPS), AN2547 (PKS) |
|  |  | 3 | down-regulated in NaCl stress (control) | AN2545 (NRPS), AN2547 (PKS) |
|  |  | 3 | down-regulated in h-H_2_O_2_ stress (*ΔatfA*) | AN2545 (NRPS), AN2547 (PKS) |
|  |  | 3 | down-regulated in tBOOH stress (*ΔatfA*) | AN2545 (NRPS), AN2547 (PKS) |
|  |  | 3 | down-regulated in diamide stress (*ΔatfA*) | AN2545 (NRPS), AN2547 (PKS) |
|  |  | 3 | down-regulated in NaCl stress (*ΔatfA*) | AN2545 (NRPS), AN2547 (PKS) |
|  |  |  |  |  |
| AN1680 cluster | 4 | 3 | up-regulation in diamide stress (control) | AN1680 (NRPS), AN1678 (TF) |
|  |  |  |  |  |
| AN2924 cluster^3^ | 4 | 4 | down-regulated in MSB stress (control) | AN2924 (NRPS) |
|  |  | 4 | down-regulated in diamide stress (control) | AN2924 (NRPS) |
|  |  | 4 | down-regulated in diamide stress (*ΔatfA*) | AN2924 (NRPS) |
|  |  |  |  |  |
| AN8209 (wA) cluster^3^ | 4 | 3 | down-regulated in MSB stress (control) | AN8209 (PKS) |
|  |  |  |  |  |
| AN12331 cluster, AN7838 (AN12331) cluster^3,4^ | 4 | 3 | down-regulated in l-H_2_O_2_ stress (control) |  |
|  |  |  |  |  |
| Microperfuranone (mic) cluster^3^ | 3 | 3 | down-regulated in MSB stress (control) | AN3396 (NRPS) |
|  |  | 3 | down-regulated in h-H_2_O_2_ stress (*ΔatfA*) | AN3396 (NRPS) |
|  |  | 3 | down-regulated in diamide stress (*ΔatfA*) | AN3396 (NRPS) |
|  |  |  |  |  |
| AN6236 cluster^3^ | 3 | 3 | up-regulation in tBOOH stress (control) | AN6236 (NRPS) |
|  |  | 2 | up-regulation in tBOOH stress (*ΔatfA*) | AN6236 (NRPS) |
|  |  | 2 | down-regulated in MSB stress (control) | AN6236 (NRPS) |
|  |  | 2 | down-regulated in NaCl stress (control) | AN6236 (NRPS) |
|  |  | 3 | down-regulated in l-H_2_O_2_ stress (*ΔatfA*) | AN6236 (NRPS) |
|  |  |  |  |  |
| AN10486 cluster | 3 | 2 | up-regulation in MSB stress (control) | AN10486 (NRPS), AN10491 (TF) |
|  |  | 2 | up-regulation in tBOOH stress (control) | AN10486 (NRPS), AN10491 (TF) |
|  |  | 2 | up-regulation in MSB stress (*ΔatfA*) | AN10486 (NRPS), AN10491 (TF) |
|  |  | 2 | up-regulation in tBOOH stress (*ΔatfA*) | AN10486 (NRPS), AN10491 (TF) |
|  |  | 2 | down-regulated in diamide stress (*ΔatfA*) | AN3911 (TF), AN10491 (TF) |
|  |  |  |  |  |
| No PKS/NRPS backbone 4 cluster^3,4^ | 3 | 2 | down-regulated in diamide stress (control) |  |
|  |  |  |  |  |
| ivo cluster^3^ | 2 | 2 | up-regulation after *atfA* deletion | AN10576 (NRPS) |
|  |  | 2 | up-regulation in l-H_2_O_2_ stress (*ΔatfA*) | AN10576 (NRPS) |
|  |  | 2 | down-regulated in tBOOH stress (*ΔatfA*) | AN10576 (NRPS) |
|  |  | 2 | down-regulated in diamide stress (*ΔatfA*) | AN10576 (NRPS) |
|  |  | 2 | down-regulated in NaCl stress (*ΔatfA*) | AN10576 (NRPS) |

^1^ - Only genes of clusters determined by either manually or experimentally were involved in the analysis [67].

^2^ - Strains and stress conditions are described in Table 1

^3^ - There is no gene encoding transcription factor among the manually/experimentally/annotated genes of the cluster

^4^- There is no gene encoding polyketide synthase, non-ribosomal peptide synthase, prenylt ransferase or terpene synthase among the manually/experimentally/annotated genes of the cluster.
