# Supplementary material for: Core oxidative stress response in Aspergillus nidulans
Source: BMC Genomics. 2015 Jun 27;16(1):478. doi: 10.1186/s12864-015-1705-z (PMC4482186; doi:10.1186/s12864-015-1705-z)
Supplement: Additional file 10: Table S10. — Primer pairs used in this study. [file 12864_2015_1705_MOESM10_ESM.doc]

**Supplementary Table 1** Primer pairs used in this study

| **Gene ID,**  **annealing temperature** | **Forward primer** | **Reverse primer** |
| --- | --- | --- |
| AN0150, 51 ºC | 5’-TACAACCACAGCAAAGATAGAC-3’ | 5’-CAAAGGCAGGAACAAGAGC-3’ |
| AN0209, 51 ºC | 5’-ATGATTGTTGTTGGCGGCG-3’ | 5’-CAGAGTCGTGTTGTGAGGC-3’ |
| AN0231, 51 ºC | 5’-ATCTCCTCTCTTCGCTCG-3’ | 5’-GGCAACAAAGTCATCCACG-3’ |
| AN0973, 51 ºC | 5’-CGACTTTCTCTCTGGATACGATG-3’ | 5’-CTGGTGACGGTAGTTGTTGTTG-3’ |
| AN1006, 52 ºC | 5’-TATGTCGTCCCAAAACCCG-3’ | 5’-TTATTCTTCGTCCGCCTCC-3’ |
| AN1007, 52 ºC | 5’-TCGTGATTGGAGAAGAGCC-3’ | 5’-CGGGTATTGAGGTAGTAGTC-3’ |
| AN1008, 51 ºC | 5´-CGCTTCTTCATCGGCATCC-3´ | 5´-CATTTTCCAGTCGGGGTGTC-3´ |
| AN1117, 52 ºC | 5’-CCATCCGTGAACATACAAGC-3’ | 5’-GAAAGCGTGAGTGATACCC-3’ |
| AN1131, 52 ºC | 5’-CACCACGCTTCCCTTCTG-3’ | 5’-CTCAAATCGCCAACCTCGC-3’ |
| AN1414, 52 ºC | 5’-CGATGCCAGTATATCCGTG-3’ | 5’-GTCAGTTCTGCTCCTTGC-3’ |
| AN1513, 51 ºC | 5´-GGGGAAGGGGTTACTTTGC-3´ | 5´-TAGAGCCACGCCAGAGATTG-3´ |
| AN1652, 51 ºC | 5´-CTCTCCTCAGCCGTCAAAC-3´ | 5´-CTCACATCCCTCCTTCACAC-3´ |
| AN2072, 51 ºC | 5´-ACCTGCTGGAACATATTTACC-3´ | 5´-CAACTGGCTGAACCTGAAC-3´ |
| AN2572, 52 ºC | 5’-CCAATCACACTACTGCCTAC-3’ | 5’-TGCTCTTATCCGTCCACTTG-3’ |
| AN2846, 51 ºC | 5´-gctaaagggcaaagttatcc-3´ | 5´-agggttcgcattatctcc-3´ |
| AN3223, 51 ºC | 5’- CGCCTTCCACCAGACCTTC-3’ | 5’- CAACTCAACCTCCCCCTCC-3’ |
| AN3230, 51 ºC | 5´-CAGAGGTTTCAAGTCCCGC-3´ | 5´-TCGTCCACATCGTCAGCAC-3´ |
| AN3632, 51 ºC  , 51 ºC | 5´-AGTGCTCATCTCGCTACAGTC-3´ | 5´-AAATCCGCCCTTCGTCTCTTG-3´ |
| AN3806, 51 ºC | 5’-GACTTCTCGCTGTTTCCGC-3’ | 5’-CTTTCTCCGTTTCCTCCCTG-3’ |
| AN4271, 51 ºC | 5´-ATGGTGCTCTCAGTTTGGTTG-3´ | 5´-GTGTCAATGCCCGTCGTTTTG-3´ |
| AN4282, 52 ºC | 5’-CGATACGACCCCAGTGATG-3’ | 5’-GACCCCAGTTTTCCATAGC-3’ |
| AN4376, 51 ºC | 5’-CGGCAAGTTCACCTACATCC-3’ | 5’-ACCCTCAGCAATATAGCGG-3’ |
| AN4562, 51 ºC | 5’-TACCCCTACCCAGAACAG-3’ | 5’-CCTTGGAAATCATCGCCGTG-3’ |
| AN4809, 55 ºC | 5’-GACGCCGCTCCTATTCTCTG-3’ | 5’-ATTGCCTCTCGCTGGGTTAG-3’ |
| AN4863, 51 ºC | 5’-CGTGGCTCTTTGGTTCATCC-3’ | 5’-ACTCTTGCGTCGGGTCATC-3’ |
| AN4871, 52 ºC | 5’-TGGTCACCAGGCGAATCTC-3’ | 5’-CGGGACGAAGGATCATACG-3’ |
| AN5035, 51 ºC | 5´-TGTGATTCCTGTGGTGTCTG-3´ | 5´-CTCCTCGCCTGTTATTTTGC-3´ |
| AN5046, 53 ºC | 5’-CAATTCTCCGCCATCGTCC-3´ | 5’-GCACCAAAGATACCACCAAG-3’ |
| AN5144, 52 ºC | 5’-GCAAGCCGATGACCGAAAG-3’ | 5’-GCAAACTGTGGAGACGAAGG-3’ |
| AN5170, 51 ºC | 5’-TGGCTTTGATGCTGGAATTG-3’ | 5’-TGTCGTTGTTGTTGTCGTCG-3’ |
| AN5634, 55 ºC | 5’-GGTGGTCTTACTGCTGTGATG-3’ | 5’-CAATGGTGGATGTGATGAGGG-3’ |
| AN5660, 51 ºC | 5´-AACAGCAGCAGCACTTCTAC-3´ | 5´-ACCCACAACACCAATGACC-3´ |
| AN5781, 55 ºC | 5’-ATTCACCGACCCTCAAACCC-3’ | 5’-CGCTCAGATGCCCAGAACTTG-3’ |
| AN5862, 51 ºC | 5´-TTTGTTGTGGAATGGCTGGG-3´ | 5´-ATAGGCATCTCTCTGGGTC-3´ |
| AN5885, 51 ºC | 5´-CCACCCCAGCAGATTATTAC-3´ | 5´-TCCCCTTGATTCCCATTCC-3´ |
| AN5953, 51 ºC | 5’-ACCATCACCTTCGCATCAC-3’ | 5’-TCCCAGACAAAAGTTCCAACG-3’ |
| AN6045, 51 ºC | 5’-CTATGACCTGCGATGGCTG-3’ | 5’-TATTGGGGACGGAAGTTGC-3’ |
| AN6438, 51 ºC | 5’-TCCAGCCGAGAAGATACC-3’ | 5’-GTCCATAAGTTGTTGCCGC-3’ |
| AN6500, 51 ºC | 5´-GTCACCATCACCAAGAAGCC-3´ | 5´-TCACACGGGAAACAGCCTC-3´ |
| AN6542, 51 ºC | 5’-GAAGTCCTACGAACTGCCTGATG-3’ | 5’-AAGAACGCTGGGCTGGAA-3’ |
| AN6642, 51 ºC | 5´-GAGAGGAGACCGAACAGAAG-3´ | 5´-TTGCGATGATAAGGACCAGC-3´ |
| AN6700, 51 ºC | 5’- CCTATTCCCGAGCAAGTTC-3’ | 5’- TGATGTTCCTGACGATGGC-3’ |
| AN6838, 51 ºC | 5’-CGGAAACTCGCCGTCAATAT-3’ | 5’-GGGCAAACCCGACAATAA-3’ |
| AN7103, 51 ºC | 5’-GAGTGGGACGGGAAGAATG-3’ | 5’-GGTTACGATGGGAGACAGC-3’ |
| AN7204, 51 ºC | 5´-CCTCTTCTACTTTGCCCTG-3´ | 5´-TCTCGCTCTGTGTTGTTTGTG-3´ |
| AN7513, 51 ºC | 5’- TCTTTACCTTTCGCCTGACC-3’ | 5’- ACCTCGCCATTGCTGTTTG-3’ |
| AN7539, 52 ºC | 5’-CTGAGAAGCGGCAAAGCG-3’ | 5’-AGAGAAGACCAACGGAGC-3’ |
| AN7657, 51 ºC | 5’-CACCTTGGCTTTCTTCTCC-3’ | 5’-TCACTTCGCTCCTCATCAG-3’ |
| AN7666, 51 ºC | 5’-TCTCTTGCGATTTGATACCC-3’ | 5’-GTTTCTGCGACGATGATGTG-3’ |
| AN7884, 51 ºC | 5’- AATGCTTCTGCTCTGTCGTC-3’ | 5’- AATGCTTCTGCTCTGTCGTC-3’ |
| AN7896, 51 ºC | 5’-GAGACGGAGCAAAAGAGAC-3’ | 5’-CTGTGAAAGTGGATGTGATGG-3’ |
| AN7902, 51 ºC | 5´-AGAAGCGTTTGGAGAGTATCG-3´ | 5´-TTTGTGCGTGTAGTGAGGG-3´ |
| AN7903, 51 ºC | 5´-GCGTCCCGTCACAATAAGG-3´ | 5´-GAGAATCCACCACCACACC-3´ |
| AN8012, 51 ºC | 5´-AGAAGGAACTGAGCGAGGAGC-3´ | 5´-TGATGGCAATGAGACCGACCC-3´ |
| AN8241, 53 ºC | 5’-CGCAGAAGCCAAATCCAA-3’ | 5’-GAAGGCACCCCAAGAAAAGTC-3’ |
| AN8269, 55 ºC | 5’-TCCCGATGCCGAGAACAAG-3’ | 5’-GAAACCGACACCAAACTGACC-3’ |
| AN8637, 52 ºC | 5’-CAAACGCTCCGCCATCTA C-3’ | 5’-CTTGAGGTGCCCGAATGT C-3’ |
| AN8683, 51 ºC | 5´-AAGTATGGAAGAGGCTGGG-3´ | 5´-TGCTGTTTGGTATGGAGGG-3´ |
| AN8803, 51 ºC | 5’-CAACAAGGGCAACAGCAAC-3’ | 5’-GAGGACAGCAACATCAAGC-3’ |
| AN9339, 52 ºC | 5’-CCGAGCCCGACAACACTTAC-3’ | 5’-GTTCAGCGACGACAATGACG-3’ |
| AN10012, 51 ºC | 5´-CCACGGGAGAGGAAGTTGAC-3´ | 5´-CACGGGGCTATCGGAAGAAG-3´ |
| AN10371, 51 ºC | 5´-CATACAACATTGGGTCTCGC-3´ | 5´-GATTTGGCATTATCGTCGGG-3´ |
| AN10533, 51 ºC | 5´-TAACCGAAAGCCCTCATTCTC-3´ | 5´-ACCAACCCAAACCTTGTCTTC-3´ |
| AN10842, 51 ºC | 5´-CACTCACCCCTCTTTCTCC-3´ | 5´-ACATTCCTCCCCCGTAAAC-3´ |
